# *Borderline Personality Disorder and Emotion Dysregulation*: A new journal to meet a growing need

**DOI:** 10.1186/2051-6673-1-1

**Published:** 2014-04-10

**Authors:** Martin Bohus, Christian Schmahl, John M Oldham

**Affiliations:** Department of Psychosomatic Medicine and Psychotherapy, Central Institute of Mental Health, Medical Faculty Mannheim, Heidelberg University, Mannheim, Germany; The Menninger Clinic and Baylor College of Medicine, Houston, TX USA

Borderline Personality Disorder (BPD) has become a major focus of interest in psychiatric and psychological research over the last two decades. BPD was once considered to be a disorder with a poor prognosis that was persistent and disabling, but research has shown that it can be effectively treated and that substantial numbers of patients with BPD improve dramatically over time [1, 2]. New findings have emerged from psychometrics to genetics, greatly increasing our knowledge of the neurobiology and pathoetiology of BPD. We have often been approached by colleagues, e.g. at scientific meetings, that a journal with a specific focus on BPD and its most central feature emotion dysregulation, covering the broad and expanding scope of the field is urgently needed. Therefore, we felt the time was ripe to launch a new journal devoted to BPD. Our new open-access journal *Borderline Personality Disorder and Emotion Dysregulation* (*BPDED*; http://www.bpded.com) will provide a platform for researchers and clinicians interested in BPD and in other conditions prominently characterized by emotion dysregulation to share information and new knowledge about these important conditions.

The journal will focus on the psychological, social and neurobiological aspects of emotion dysregulation. It will cover the whole range of BPD research including, but not limited to, epidemiology, phenomenology, pathophysiology, psychological and pharmacological treatment, neurobiology, genetics, and animal models of BPD. It is important to mention that we also welcome submissions from other fields of psychiatric or psychological research, as long as there is a link to BPD or emotion dysregulation. Emotion dysregulation is at the core of BPD but also stands on its own as a major pathological component of the underlying neurobiology of various other psychiatric disorders. Prominent examples here are posttraumatic stress disorder (PTSD) and attention-deficit hyperactivity disorder (ADHD). We are very lucky to have an excellent editorial board (see also http://www.bpded.com) to assist us, all among the leading experts in BPD and neighboring disorders, who cover the whole range of this large and fascinating field of psychopathology.

Open-access publishing has several advantages: All articles are freely and universally accessible online, so that an author's work is available to readers at no cost and not limited by library budgets. This ensures that the author’s work is disseminated to the widest possible audience. Open access journals have the potential to reach a much larger set of readers than any subscription-based journal, in print and online [3]. Some studies have even suggested a correlation between open access, higher downloads and higher citations, leading to a higher Impact Factor [4, 5]. In contrast to other types of publication, authors hold copyright for their work and can grant anyone the right to reproduce and disseminate the article, provided that it is correctly cited and no errors are introduced.

*BPDED* articles will be archived in PubMed Central, and other freely accessible full-text repositories. This complies with the policies of a number of funding bodies including the Wellcome Trust, NIH and Howard Hughes Medical Institute. A country's economy will not influence its researchers' ability to access articles because resource-poor countries (and institutions) will be able to read the same material as wealthier ones. The traditional business model for scientific publishers relies on restricting access to published research, in order to recoup the costs of the publication process. This restriction of access to published research prevents full use being made of digital technologies, and is contrary to the interests of authors, funders and the scientific community as a whole.

In contrast, an open access publishing model treats publication as the last phase of the research process. Article-processing charges (APCs) cover the cost of the publication process to allow free and immediate access to the research articles. APCs ensure transparency and allow publishers to compete to provide the best service at the best price. By coupling the cost of publication to research budgets, APCs ensure that the journal publishing system can scale to cope with an ever-increasing volume of research. Several funding agencies support open-access publishing and provide budgets for APCs. Since we are aware of the fact that APCs still might discourage authors from publishing in open-access journals, we are pleased that the German Association for DBT has provided funding for approximately the first two years of the journal’s publication to cover APCs on behalf of authors who do not have another means of funding. Articles will be rapidly peer-reviewed and will be published online soon after acceptance and will later be listed in PubMed.

Taken together, we strongly believe that our new journal is an important addition to the journal landscape on borderline personality disorder. With your highly appreciated contributions as authors submitting to *BPDED* as well as your interest as readers of the articles, we are convinced to make this journal a success. We sincerely hope that it will become an integral part of research in borderline personality and emotion dysregulation.

Martin Bohus MD, Christian Schmahl MD, John M Oldham MD.

## References

[1] Lieb K, Zanarini MC, Schmahl C, Linehan MM, Bohus M (2004). Borderline personality disorder. Lancet.

[2] Leichsenring F, Leibing E, Kruse J, New AS, Leweke F (2011). Borderline personality disorder. Lancet.

[3] Suber P (2005). Open access, impact, and demand. BMJ.

[4] Hitchcock S: *The effect of open access and downloads ('hits') on citation impact: a bibliography of studies*. http://opcit.eprints.org/oacitation-biblio.html

[5] Brody T, Harnad S: *Earlier web usage statistics as predictors of later citation impact*. http://eprints.ecs.soton.ac.uk/10713/02/timcorr.htm

